# Real-World Outcomes of Atezolizumab with Bevacizumab Treatment in Hepatocellular Carcinoma Patients: Effectiveness, Esophagogastroduodenoscopy Utilization and Bleeding Complications

**DOI:** 10.3390/cancers16162878

**Published:** 2024-08-19

**Authors:** Cha Len Lee, Mark Freeman, Kelly W. Burak, Gordon T. Moffat, Conor D. J. O’Donnell, Philip Q. Ding, Hanna Lyubetska, Brandon M. Meyers, Vallerie Gordon, Ekaterina Kosyachkova, Roxana Bucur, Winson Y. Cheung, Jennifer J. Knox, Vincent C. Tam

**Affiliations:** 1 Princess Margaret Cancer Center, University of Toronto, Toronto, ON M5G1Z5, Canada; jennifer.knox@uhn.ca (J.J.K.); 2 Tom Baker Cancer Center, University of Calgary, Calgary, AB T2N4N2, Canada; vincent.tam@albertahealthservices.ca (V.C.T.); 3 Liver Unit, Divisions of Gastroenterology & Hepatology and Transplant Medicine, Departments of Medicine and Oncology, Cumming School of Medicine, University of Calgary, Calgary, AB T2N4N2, Canada; kwburak@ucalgary.ca; 4 Juravinski Cancer Center, Escarpment Cancer Research Institute, McMaster University, Hamilton, ON L8S4L8, Canada; 5 CancerCare Manitoba, University of Manitoba, Winnipeg, MB R3A1R9, Canada

**Keywords:** atezolizumab, bevacizumab, hepatocellular carcinoma, EGD, varices, GI bleeding

## Abstract

**Simple Summary:**

Our real-world multicenter retrospective analysis suggests that omitting an esophagogastroduodenoscopy (EGD) in specific hepatocellular carcinoma (HCC) patients may be a safe and cost-effective strategy for those undergoing atezolizumab with bevacizumab (A+B), without leading to an elevated risk of bleeding complications. In our study, conducted in Canadian centers during the early access period of A+B, 30% of patients did not undergo pre-treatment EGD. This clinical decision was often based on the absence of cirrhosis, significant thrombocytopenia, or a low likelihood of portal hypertension, as assessed by their physicians. Despite the absence of standardized guidelines and the use of an individualized approach to EGD screening, patients did not experience negative treatment outcomes or worse survival. Our data also indicated that bleeding complications associated with A+B treatment are predominantly non-GI in nature. There may be several reasons not to use EGD routinely in this setting, which include a balance between patient risks and healthcare resources. EGD is an invasive procedure that requires sedation and carries a small risk of complications, along with potential discomfort and anxiety. The limited availability of expert endoscopists’ time could also lead to significant delays in initiating effective therapy in the advanced HCC setting where A+B has been shown to prolong life.

**Abstract:**

The IMbrave150 trial established atezolizumab with bevacizumab (A+B) as standard care for hepatocellular carcinoma (HCC), recommending an esophagogastroduodenoscopy (EGD) within 6 months of treatment initiation to prevent bleeding from esophagogastric varices. The necessity of mandatory EGD for all patients remains unclear. We retrospectively analyzed 112 HCC patients treated with A+B at five Canadian cancer centers from 1 July 2020 to 31 August 2022. A+B was the first-line therapy for 90% of patients, with median overall survival at 20.3 months and progression-free survival at 9.6 months. There was no survival difference between patients with bleeding and those without. Before A+B, 71% (n = 79) of patients underwent an EGD within 6 months, revealing varices in 41% (n = 32) and requiring intervention in 19% (n = 15). The overall bleeding rate was 15% (n = 17), with GI-specific bleeding occurring in 5% (n = 17). In the EGD group, GI-specific bleeding was 6% (n = 5) while in the non-EGD group, it was 3% (n = 1). Non-GI bleeding was observed in 10% (n = 11) of patients. Outcomes for HCC patients treated with A+B in Canada were comparable to IMbrave150. There was no increase in GI bleeding in patients without pre-treatment EGD, possibly supporting a selective EGD approach.

## 1. Introduction

Hepatocellular carcinoma (HCC) constitutes 75–90% of primary liver cancer and ranks as the third leading cause of global cancer-related deaths [1,2]. Predictions indicate a 41% increase in global HCC mortality rates by 2040 [2,3]. HCC is a tumor characterized by high vascularization [4], and clinical trial data emphasize targeting vascular endothelial growth factor (VEGF)-mediated angiogenesis in HCC treatment [5,6,7,8]. Anti-VEGF agents including sorafenib, lenvatinib, and regorafenib have been standard systemic therapies, but they only provided a modest improvement in survival [9]. The IMbrave150 trial showed superior outcomes with atezolizumab with bevacizumab (A+B) in terms of overall survival (19.2 vs. 13.4 months), progression-free survival (6.9 vs. 4.3 months), and objective response rate (30% vs. 11%) compared to sorafenib [10,11]. Based on these results, A+B has become a recognized first-line immunotherapy-based combination for untreated locally advanced or metastatic HCC.

Approximately 80% of HCC cases occur in the presence of cirrhosis, putting individuals at risk of portal hypertension, thus complicating the management of HCC [12,13]. In the IMbrave150 trial, all participants were required to undergo esophagogastroduodenoscopy (EGD) within 6 months of starting treatment. This protocol aimed to detect and treat all esophagogastric varices to minimize potential GI-related bleeding complications related to the high-dose bevacizumab (15 mg/kg) administered to patients in the experimental arm. The IMbrave150 trial reported a higher incidence of GI bleeding in A+B patients (7%) compared to the sorafenib arm (4.5%) [10]. Similar results were recapitulated in the ORIENT-32 trial with the combination of sintilimab and IBI305, a bevacizumab biosimilar [14]. Given the increased risk of GI bleeding associated with bevacizumab despite mandated pre-treatment EGD in the IMBrave150 trial, current guidelines from the American Society of Clinical Oncology (ASCO), European Society for Medical Oncology (ESMO), American Association for Study of Liver Disease (AASLD), American Gastroenterological Association (AGA), and National Comprehensive Cancer Network (NCCN) suggest patients undergo EGD assessment within 6 months before commencing A+B [15,16,17,18].

The necessity of pre-treatment EGD for all HCC patients has been a subject of debate among some medical oncologists and hepatologists. It remains uncertain whether every patient requires a screening EGD before A+B. In cases where cirrhosis is not documented by a hepatology team and there is a low risk of portal hypertension, initiation of A+B without a recent EGD may be a justifiable approach. This practice has been adopted at a few Canadian cancer centers, mainly to avoid delays associated with waiting for an EGD, which could hinder the timely commencement of systemic therapy. Mandating EGD for patients with a low risk of portal hypertension is also not a cost-effective practice. There are alternative non-invasive methods (i.e., transient elastography and platelet count) to predict clinically significant portal hypertension (CSPH) although their use in HCC cases is not fully verified [19,20]. Primary prophylaxis for preventing variceal bleeding has evolved in the last decade, with both endoscopic band ligation and non-selective beta-blockers considered acceptable strategies in patients with cirrhosis [21,22]. To our knowledge, limited data exist regarding the safety of selective EGD in HCC patients undergoing A+B.

The primary aims of this study were to evaluate the effectiveness of first-line A+B in real-world clinical practice, the uptake of pre-treatment EGD, and treatment-related bleeding rates in HCC patients.

## 2. Materials and Methods

### 2.1. Study Design

We conducted a pooled population-based retrospective study of HCC patients who received A+B outside clinical trials. This study utilized the Hepatocellular Carcinoma Cancer Health Outcomes Research Database (HCC-CHORD) consortium, a national collaboration of oncologists at several Canadian cancer centers. Ethics approval was obtained from collaborating institutions for the HCC-CHORD project.

### 2.2. Study Population

We identified all patients with a confirmed histologic and/or radiographic diagnosis of HCC who received A+B in any line of treatment from 1 July 2020 to 31 August 2022. All patients with locally advanced, metastatic, or unresectable HCC were eligible for inclusion. Patients who received A+B were identified through cancer center pharmacy database records. Patient charts were reviewed to confirm the HCC diagnosis, and that each patient had received at least one treatment with A+B. There was no pre-planned sample size used in this study, as all patients who received at least one treatment with A+B at participating institutions during this time period were included.

### 2.3. Data Collection

Data for this study was obtained from two cancer centers in the province of Ontario, all cancer centers in Alberta, and one cancer center in Manitoba. Data collection was completed using a uniform database template across all collaborating sites. Patient demographics included ethnicity, Eastern Cooperative Oncology Group (ECOG) performance status, underlying liver disease etiology, Child-Pugh class, Barcelona Clinic Liver Cancer (BCLC) staging, tumor-related characteristics, previous locoregional treatments, routine blood results, pre-treatment EGD, and the systemic therapy received. Bleeding is defined as clinical events that occur after starting A+B and consist of a drop in hemoglobin level, need for blood transfusion, or further intervention including temporarily or permanently discontinuing A+B. The bleeding is categorized into GI-related or non-GI-related events.

### 2.4. Statistical Analysis

The primary objective was to assess the real-world effectiveness of A+B. A comparative analysis was conducted to evaluate the patients’ outcomes in this study with those observed in the IMbrave150 trial. The primary outcome was overall survival (OS), measured in months from the start date of A+B until the date of death, considering censoring at the last follow-up. Secondary outcomes included progression-free survival (PFS) and objective response rate (ORR). PFS was defined as the duration from the start of A+B to either disease progression or death. ORR was determined based on the treating physician’s assessment of radiographic imaging and/or radiologist reports as practical limitations prevented the imaging assessment with RECIST 1.1 and/or modified RECIST (mRECIST) criteria.

Patient demographics, treatment details, and toxicities were summarized using descriptive statistics. Categorical variables were presented as frequency counts and proportions, and continuous variables were presented as median and interquartile range (IQR). Pearson’s Chi-square test or Fisher’s exact test was employed to compare subgroups for categorical variables, while the Wilcoxon rank-sum test was used for continuous variables. The Kaplan-Meier method was used to assess time-to-event outcomes (i.e., PFS, OS) and statistical comparisons were made using the log-rank test. A Cox proportional hazards model was constructed, incorporating relevant clinical and pathological factors, to estimate their impact on bleeding risk and survival. All statistical tests were two-sided, and the significance level was defined a priori as <0.05. Analyses were performed using the FactoMineR package on the R software (version 4.3.0).

## 3. Results

### 3.1. Patient Population

We identified a total of 112 patients treated with A+B. Patient characteristics are shown in Table 1. The median age was 66 years, with 87% were male, and 24% were East Asian. The proportion of patients with cirrhosis was 67%, diagnosed either histologically or radiologically, including transient elastography. The predominant etiologies of liver disease were hepatitis C virus (35%) and hepatitis B virus (26%). Before A+B, 95% had an ECOG 0–1, 91% were Child-Pugh A, and 71% had BCLC stage C disease. Portal vein thrombosis was present in 37% of patients. Before A+B, 72% of patients had received locoregional therapies.

At the time of data cut-off, 72% (81) of patients had discontinued A+B. Reasons for treatment discontinuation included disease progression (37%), patient choice (26%), toxicity (18.5%), and cancer-related death (18.5%). In terms of line of therapy, 90% (101) received A+B as first-line, 9% (10) as second-line, and 1% (1) as a third-line treatment option. All systemic treatments administered in this study population are shown in Table 2.

### 3.2. Effectiveness of Atezolizumab and Bevacizumab

**Figure 1:**
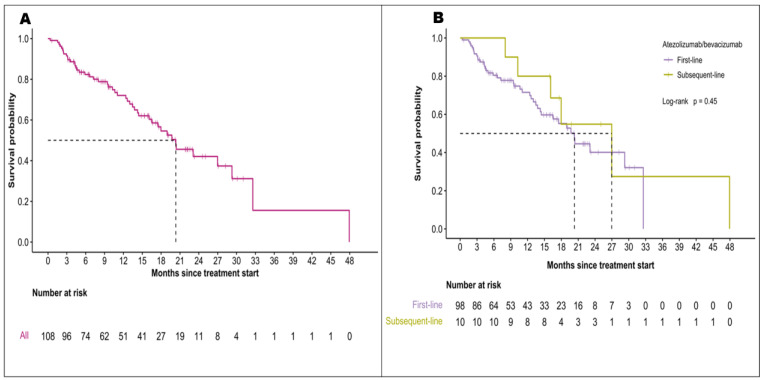
Overall survival in patients treated with atezolizumab with bevacizumab for (**A**) the entire study population and (**B**) different lines of treatment.

**Figure 2:**
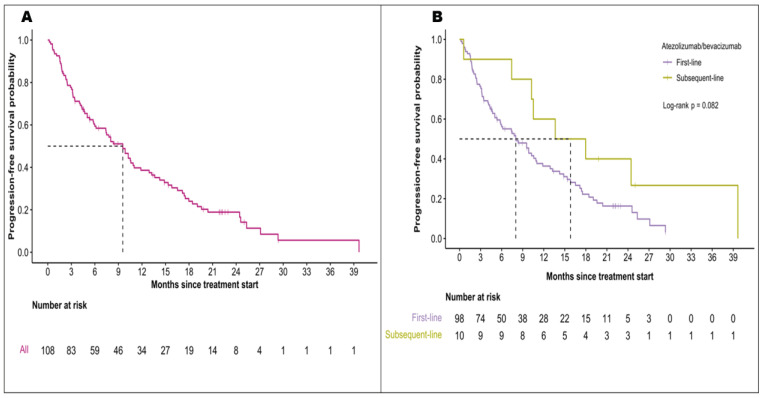
Progression-free survival in patients treated with atezolizumab with bevacizumab for (**A**) the entire study population and (**B**) different lines of treatment.

Table 3 illustrates a comparative analysis of A+B treatment efficacy between this study and the IMbrave150 trial. During a follow-up period of 10.4 months (95% CI, 0.4–47.9), the overall median OS was 20.3 months (95% CI, 16.5-*NR*) [Figure 1A]. For the patients treated with A+B as first-line, the median OS was 20.3 months (95% CI, 14.4-*NR*). For patients who received A+B as a second or third-line treatment, the median OS was 27.0 months (95% CI, 16.1-*NR*; *p* = 0.45) [Figure 1B]. The overall median PFS was 9.6 months (95% CI, 6.1–11.9) [Figure 2A]. In the first-line treatment cohort, PFS was 8.0 months (95% CI, 5.8–11.0), and for subsequent lines was 15.8 months (95% CI, 10.3-*NR*) (*p* = 0.082) [Figure 2B]. Baseline Albumin-bilirubin (ALBI) grade 1 was significantly associated with prolonged OS but did not seem to influence PFS. The OS and PFS did not differ by liver disease etiologies or the presence of portal vein tumor thrombosis [Figures S1 and S2].

The overall ORR was 36%, including a complete response in 1 patient (1%). Overall, the disease control rate is 77%. This is consistently observed whether A+B was employed as a first-line (77%) or subsequent-line treatment (78%) (*p* = 0.16).

### 3.3. EGD Utilization and Esophagogastric Varices

Among 112 patients who received A+B, 71% (79) underwent EGD within 6 months before starting A+B. The remaining 29% (33) of patients proceeded with A+B without an EGD. The main reasons for not undergoing pre-treatment EGD are listed in Table 1. The reasons for not conducting EGD in 26 patients were not documented.

More patients in the EGD group had cirrhosis (68%) compared to the non-EGD group (32%), although this difference is not statistically significant (*p* = 0.45). Table 4 compares patients who underwent EGD with those who did not. Patients in the EGD group had a lower median platelet count than the non-EGD group (162 × 10^9^/L vs. 189 × 10^9^/L, *p* = 0.17). Only 5% (6 out of 112) of patients had a platelet count below 150 × 10^9^/L, and 2% (2 out of 112) had a platelet count below 100 × 10^9^/L.

Among the 79 patients in the EGD group, 41% (32) of patients were found to have varices, and 19% (15) had received intervention for varices before A+B, either through endoscopic band ligation and/or oral beta-blockers.

### 3.4. Bleeding Events during A+B Treatment

A non-significant trend suggests that patients with evidence of varices experienced more bleeding during A+B (39% vs. 10%, *p* = 0.07). Patients with high-risk varices that required intervention developed more bleeding events compared to those not needing variceal treatment (47% vs. 11%, *p* = 0.003). The overall bleeding rate was 15% (17), with the GI-specific bleeding rate being relatively low at 5% (6). In the EGD group, GI-specific bleeding was 6% (5), while in the non-EGD group, it was 3% (1), attributed to diverticular bleeding. The rate of non-GI bleeding events was 10% (n = 11; 6 epistaxis, 1 ecchymosis, 1 periodontal, and 3 unspecified).

There was a significant association between bleeding risk and patients with hepatitis B virus and those with a history of multiple TACE procedures (both *p* = 0.03) [Table S1]. Patients with bleeding events also exhibited a lower platelet count of median 127 × 10^9^/L (range 84–220), whereas those without bleeding had a platelet count of 188 × 10^9^/L (range 49–574); *p* = 0.001.

Bleeding complications did not impact patient survival. Median OS was 20.3 months (95% CI, 13.0-*NR*) among patients with bleeding, compared to 19.7 months (95% CI, 16.5-*NR*) for those without bleeding; *p* = 0.78. PFS also did not differ between patients with bleeding and those without (13.8 vs. 14.8 months; *p* = 0.95) [Table 3].

## 4. Discussion

Our results provide evidence that the real-world effectiveness of first-line A+B in Canadian clinical practice appears similar to the outcomes observed in the IMbrave150 trial [11]. The higher PFS and ORR observed in our study may be attributed to the physician assessments documented in clinical notes, as opposed to the independently assessed RECIST 1.1 and HCC-specific mRECIST criteria used in the IMbrave150 trial. The significantly longer PFS seen in our patients receiving subsequent treatments may be due to the supportive care provided by the Canadian health system. This underscores the importance of optimal supportive management for patients even after treatment cessation. The median OS in this study is higher than other real-world studies (Casadei-Gardini et al., Fulgenzi et al., and D’Alessio et al.) which reported median OS in the range of 14.9 to 16.4 months [23,24,25]. Discrepancies may arise from variations in study populations in terms of HCC stage, underlying liver disease etiologies, liver function, and treatment availability. In contrast to Casadei-Gardini and Fulgenzi et al., this study recruited fewer patients with prior TACE (26%), a procedure recognized for its potential adverse impact on liver function. D’Alessio et al. involved 216 patients across Europe, the United States, and parts of Asia, and reported the lowest median OS of 14.9 months among all the studies conducted in a real-world setting [23,26]. This is likely related to the higher proportion of patients with Child-Pugh B liver function (24%), compared to 9% in our study [23]. Child-Pugh A patients showed superior OS with A+B than patients with Child-Pugh B (16.8 vs. 6.7 months, respectively, *p* = 0.0003) [23]. Our study also had a slightly higher percentage of patients with viral etiology (61% vs. 55% in Casadei-Gardini et al.) [25]. This could serve as a contributing factor, since patients with viral etiology may derive more benefit from immune checkpoint inhibition, as indicated by subgroup analyses [27]. A meta-analysis by Kulkarni et al. which included 5400 patients in a real-world setting demonstrated A+B effectiveness across liver disease etiologies [26].

Despite our selective approach, with 71% of patients undergoing an EGD, our GI-specific bleeding incidence was comparable to the patients who received A+B in the IMbrave150 trial (5% vs. 7%) [11]. This is also comparable to the GI bleeding rates seen in other clinical trials of combination anti-VEGF agents plus immune checkpoint inhibitors. In the ORIENT-32 trial, patients treated with sintilimab in combination with IBI305, a biosimilar to bevacizumab, had a total GI bleeding rate of 5% [14]. In trials of tyrosine kinase inhibitors (TKIs) plus immune checkpoint inhibitors, such as COSMIC-312, patients treated with atezolizumab plus cabozantinib had a GI bleeding rate of less than 1% [28]. In the LEAP-002 trial, a pre-treatment EGD was mandated within three months of initiating therapy with lenvatinib plus pembrolizumab, where the incidence of upper GI bleeding was reported to be less than 1% [29,30]. In the CARES-310 trial, the combination of rivoceranib plus camrelizumab in HCC patients was associated with an upper GI bleed incidence of 3% [31].

Comparison between groups with and without EGD in our study revealed no significant differences in bleeding rates or baseline characteristics. No negative impact on treatment outcomes was observed among 29% of patients who did not undergo EGD. We observed a higher rate of varices detection (41% vs. 26%) and treatment (19% vs. 11%) than the IMbrave150 trial, reflecting a real-world treatment setting involving more advanced diseases and cirrhosis [10]. Although more bleeding occurred among patients who had received varices treatment (*p* = 0.003), it is plausible that the observed bleeding may be attributed to early bleeding from post-banding ulcers. However, we lacked specific details of patients who underwent endoscopic band ligation.

High-risk varices defined by size > 5 mm or red markings (indicating a thin wall) should receive prophylaxis, with either endoscopic band ligation or non-selective beta-blockers, to prevent bleeding consequences. Both options are considered acceptable standard treatment, as randomized clinical trials did not establish a preferred strategy [17,21,22,32,33]. The PREDESCI trial, however, favored the beta-blockers option for preventing hepatic decompensation, which subsequently reduces the incidence of ascites, varices, and variceal bleeding [21]. Another study showed that the benefit of endoscopic band ligation in preventing variceal bleeding and improving OS is consistently observed across the whole HCC population, with beta-blockers being more effective in patients with BCLC stage C/D [34].

Non-invasive tools such as liver stiffness measurement (LSM) using transient elastography have reshaped the management of chronic liver disease patients [19]. Based on BAVENO VI criteria, cirrhotic patients with LSM < 20 kPa and a platelet count > 150 × 10^9^/L are unlikely to have high-risk varices (3%) and can therefore forgo EGD screening [20]. A meta-analysis involving 7387 patients with cirrhosis confirmed the high sensitivity (97%) for BAVENO VI criteria in detecting high-risk varices [35]. This meta-analysis reported a lower prevalence of high-risk varices of 14%, as compared to 26% observed in the IMbrave150 trial, although not all varices identified were classified as high-risk [10]. The utilization of BAVENO VI criteria in the HCC cases is still limited, with studies reporting varying false negative rates. Wu et al. recently validated these criteria in a prospective study involving advanced HCC patients, demonstrating a 21% reduction in unnecessary EGD [36]. In this analysis, BAVENO VI criteria demonstrated a sensitivity of 87%, a negative predictive value of 85%, and a false negative of 3.5% [36]. In contrast, a retrospective French study involving 185 HCC patients demonstrated a minimum 8% false negative rate in applying the BAVENO VI criteria to exclude high-risk varices. The rate was higher in subgroups with larger infiltrative tumors (>5 cm) or main portal vein thrombosis [37]. Otherwise, this study reported comparable rates of sensitivity at 93% and negative predictive value at 92% [37].

The updated BAVENO VII consensus from 2022 continues to rely on the BAVENO VI criteria for determining which cirrhotic patients can safely avoid endoscopic investigation [38]. In HCC patients, future research is needed to establish specific cut-off platelet count and LSM values that would warrant an EGD, particularly in cases with confounding risk factors such as large-sized or multinodular tumors, portal vein thrombosis, or previous varices. BAVENO VII suggests beta-blockers as a primary option for variceal bleeding prophylaxis in cirrhotic patients, and it may be reasonable to initiate beta-blockers in low-risk individuals [33]. EGD should be contemplated for patients deemed high risk or those needing endoscopic band ligation due to active bleeding, contraindications, or beta-blocker intolerance. This approach is preferred because endoscopic band ligation carries the risk of bleeding from post-banding ulcers and may necessitate multiple sessions for complete variceal eradication, potentially causing treatment delays. It is important to note that hypertension occurred in 28% of patients (39% Grade 3/4) undergoing A+B [11,26]. Ideally, oncologists should opt for beta-blockers over alternative anti-hypertensive drugs as this choice not only manages the treatment side effects but also provides preventive benefits against variceal bleeding.

The IMbrave150 trial indicated that most treatment-related bleeding events occurred later, often coinciding with disease progression [11]. Rather than strictly performing EGD before starting A+B, screening EGD could be conducted at different intervals during treatment based on personalized clinical risk features. Protocols allowing EGD within 12 months, rather than 6 months, are less burdensome on the healthcare system. PFS and OS did not differ significantly between our patients who experienced bleeding and those who did not. While it is reassuring that bleeding events do not significantly affect survival in both EGD and non-EGD groups, it is crucial to note that most of the bleeding events in our study were non-GI in nature. A significantly increased bleeding risk was observed in patients with prior multiple TACE procedures or hepatitis B virus, however, the limited sample size in both cohorts warrants caution in drawing definitive conclusions. No significant correlations in bleeding incidence were found concerning baseline tumor factors.

Our study’s main limitations include its retrospective design and small sample size, particularly in the non-EGD group, which may introduce biases and limit the establishment of causal relationships. Budgetary and logistical constraints also restricted the objective assessment of imaging according to RECIST 1.1 criteria. Our data analysis is further limited by the etiology of GI bleeding and the absence of time-dependent variables such as varices grade, severity of bleeding events, and the use of concurrent anticoagulants and beta-blockers before EGD. Medical records frequently lacked documentation explaining why some patients did not undergo EGD. Physicians likely selected candidates for EGD based on clinical risk factors for portal hypertension and bleeding. When documented, the most common reason for forgoing EGD was a collaborative decision with hepatologists due to the patient’s clinical absence of cirrhosis or low likelihood of portal hypertension. Despite these considerations, insights from our retrospective analysis of 33 patients without EGD suggest that selective EGD in a well-defined HCC cohort may be a reasonable approach.

## 5. Conclusions

The effectiveness of A+B and the associated risk of GI bleeding in real-world practice appear similar to those observed in the IMbrave150 trial. Targeted EGD screening does not seem to adversely affect treatment outcomes or increase the risk of GI bleeding. We are conducting a prospective study (EVBER-HCC: Evaluating Gastrointestinal Bleeding Risks and EGD Requirement in Low-Risk HCC Patients Undergoing Atezolizumab and Bevacizumab; University Health Network institutional authorization CAPCR#:23-5915) to validate this finding. This study employs non-invasive stratification tools, such as transient elastography and platelet count, to identify HCC patients without hepatic decompensation and with a low risk of portal hypertension who can safely avoid pre-treatment EGD.

## Figures and Tables

**Table 1:** Patient Characteristics.

Characteristic	Total (n = 112)
**Median Age (IQR), years**	66 (24–85)
**Sex**	
Male	97 (87%)
Female	15 (13%)
**Ethnicity**	
East-Asian	27 (24%)
Other	85 (76%)
**Province**	
Alberta	60 (54%)
Ontario	48 (43%)
Manitoba	3 (3%)
Unknown	1
**ECOG performance score**	
0	53 (47%)
1	54 (48%)
2	4 (4%)
3	1 (1%)
**Child-Pugh score**	
A	99 (91%)
B	10 (9%)
Unknown	3
**Albumin-bilirubin (ALBI) grade**	
1	50 (45%)
2	60 (54%)
3	1 (1%)
Unknown	1
**Barcelona Clinic Liver Cancer (BCLC) stage**	
A	7 (6%)
B	26 (23%)
C	79 (71%)
**Liver disease etiology**	
Hepatitis C	37 (35%)
Hepatitis B	28 (26%)
Alcohol use	25 (23%)
NASH	17 (16%)
Other	5
**Cirrhosis**	
Yes	75 (67%)
No	37 (33%)
**Liver resection**	29 (28%)
**Prior locoregional therapy**	
Yes	57(72%)
No	22 (28%)
Unknown	33
**Type of locoregional therapy**	
Radiofrequency Ablation (RFA)	33 (29%)
Stereotactic Body Radiation Therapy (SBRT)	17 (15%)
y-90 Radioembolization	6 (5.4%)
Transarterial embolization (TAE)	4 (3.6%)
Transarterial chemoembolization (TACE)	29 (26%)
1	11 (38%)
2	14 (48%)
3	4 (14%)
**Tumor histology**	
HCC	73 (65%)
Mixed HCC-CCA	3 (3%)
No biopsy/Unknown	36 (32%)
**Size of largest lesion, cm**	
Median (IQR)	3.8 (0.0–18.0)
Unknown	16
**Tumor distribution**	
Unilobar	55 (50%)
Bilobar	55 (50%)
Unknown	2
**Macrovascular invasion**	
Yes	41 (37%)
No	70 (63%)
Unknown	1
**Presence of lymph node or extrahepatic metastasis**	
Lymph node involvement	31 (28%)
Extrahepatic metastasis	41 (37%)
**Completed EGD within 6 months**	
Yes	79 (71%)
No	33 (29%)
**Reasons for no EGD**	
No radiological evidence of cirrhosis or varices	4 (12%)
Limited EGD availability	2 (6%)
Patient refusal (with EGD within 7 months)	1(3%)
Unknown	26 (79%)
**Varices**	
Detected on EGD	32 (41%)
Required intervention before A+B	15 (19%)
No previous EGD/Unknown	34
**Baseline AFP level, µg/L**	
Median (IQR)	47.0 (1.0–86,709)
Unknown	2
**Baseline platelet count, ×10^9^/L**	
Median (IQR)	177.0 (49.0–574.0)
Unknown	1
**Reasons for A+B discontinuation**	
Disease progression	30 (37%)
Patient choice	21 (26%)
Toxicities	15 (18.5%)
Death	15 (18.5%)

IQR: interquartile range; NASH: non-alcoholic steatohepatitis; HCC: hepatocellular carcinoma; CCA: cholangiocarcinoma; EGD: esophagogastroduodenoscopy; AFP: alpha-fetoprotein.

**Table 2:** Lines of systemic treatments.

Characteristic	Total (n = 112)
**First-line regimen (n = 112)**	
A+B	101(90%)
Lenvatinib	6 (5%)
Sorafenib	4 (4%)
Chemotherapy	1 (1%)
**Second-line regimen (n = 39)**	
Lenvatinib	25 (64%)
A+B	10 (26%)
Sorafenib	2 (5%)
Chemotherapy	2 (5%)
**Third-line regimen (n = 7)**	
Regorafenib	2 (29%)
Lenvatinib	2 (29%)
A+B	1 (14%)
Cabozantinib	1 (14%)
Sorafenib	1 (14%)
**A+B prior to TKIs**	101 (90%)
**TKIs prior to A+B**	11 (10%)

A+B: atezolizumab with bevacizumab; TKIs: tyrosine kinase inhibitors.

**Table 3:** Comparison of real-world effectiveness of atezolizumab with bevacizumab between our study and the IMbrave150 trial. Our study utilized physician-assessed progression, while the IMbrave150 trial employed RECIST 1.1 criteria.

Characteristics	This Study (n = 112)			IMbrave150 [11] (n = 336)
Median follow-up period (95% CI), months	10.4 (0.4–47.9)			17.6 (0.1–28.6)
Median treatment duration	6.4 (0.2–29.8)			8.4 (3.5–18.3)
Median OS (95% CI) months	20.3 (16.5-*NR*)			19.2 (17.0–23.7)
18-months OS	55%			52%
Median OS (patients with bleeding vs. without bleeding)	20.3 (13.0-*NR*) vs. 19.7 (16.5-*NR*); *p* = 0.86			
Median PFS * (95% CI), months	9.6 (6.1–11.9)			6.9 (5.7–8.6)
Median PFS (patients with bleeding vs. without bleeding)	10.3 (5.0–27.1) vs. 9.6 (7.4–13.6) *p* = 0.95			
ORR *	Overall (n = 112)	First-line (n = 101)	Subsequent-line (n = 11)	Overall (n = 336)
Complete response no. (%)	1(1%)	0 (0%)	1 (11.1%)	25 (8%)
Partial response no. (%)	36 (35.0%)	33 (35.1%)	3 (33.3%)	72 (22%)
Stable disease no. (%)	42 (40.8%)	39 (41.5%)	3 (33.3%)	144 (44%)
Progressive disease no. (%)	24 (23.3%)	22 (23.4%)	2 (22.2%)	63(19%)
Could not be evaluated/Missing no.	9	7	2	22 (7%)

OS: overall survival, PFS: progression-free survival; *NR*: not reached; ORR: objective response rate. * PFS and ORR data are based on physician-reported assessment of response and progression.

**Table 4:** Comparison of baseline clinical characteristics and bleeding rates in patients with and without pre-treatment esophagogastroduodenoscopy (EGD).

		EGD within 6 Months		
Characteristic	Total (n = 112)	Yes (n = 79)	No (n = 33)	*p*-Value
**Cirrhosis**	75	51(68%)	24(32%)	0.45
**Platelet count, ×10^9^/L**				
Median (IQR)	177 (49–574)	162 (65–574)	189 (49–486)	0.17
**Albumin, g/L**				
Median (IQR)	39.0 (24–421)	39 (26–421)	39 (24–48)	0.70
**INR**				
Median (IQR)	1.1 (0.9–1.8)	1.1(0.9–1.4)	1.1 (0.9–1.8)	0.86
**Total Bilirubin, µmol/L**				
Median (IQR)	15 (3–72)	15 (3–72)	16 (3–57)	0.56
**Size of largest tumor, cm**				
Mean ± SD	96	6.0 ± 4.3	5.7 ± 4.8	0.56
Unknown	16			
**Macrovascular invasion**	41	12 (15%)	28 (85%)	0.88
Unknown	1			
**Bleeding events**	17	14 (18%)	3 (9%)	0.24
GI-specific bleeding	6	5 (6%)	1 (3%)	

IQR: interquartile range; SD: standard deviation.

## Data Availability

Research data supporting this publication are included within this article.

## Supplementary Materials

The following supporting information can be downloaded at: https://www.mdpi.com/article/10.3390/cancers16162878/s1, Figure S1: Overall Survival in Patients Treated with Atezolizumab with Bevacizumab Stratified by (A) ALBI grade (B) Liver etiology and (C) Portal vein tumor thrombosis; Figure S2: Progression-Free Survival in Patients Treated with Atezolizumab with Bevacizumab Stratified by (A) ALBI grade (B) Liver etiology and (C) Portal vein tumor thrombosis; Table S1: Impact of clinical characteristics on bleeding risk.
