# Supplementary material for: Real-World Outcomes of Atezolizumab with Bevacizumab Treatment in Hepatocellular Carcinoma Patients: Effectiveness, Esophagogastroduodenoscopy Utilization and Bleeding Complications
Source: Cancers (Basel). 2024 Aug 19;16(16):2878. doi: 10.3390/cancers16162878 (PMC11352899; doi:10.3390/cancers16162878)
Supplement: Supplementary file 1 [file cancers-16-02878-s001.zip › cancers-3107230-supplementary.pdf]

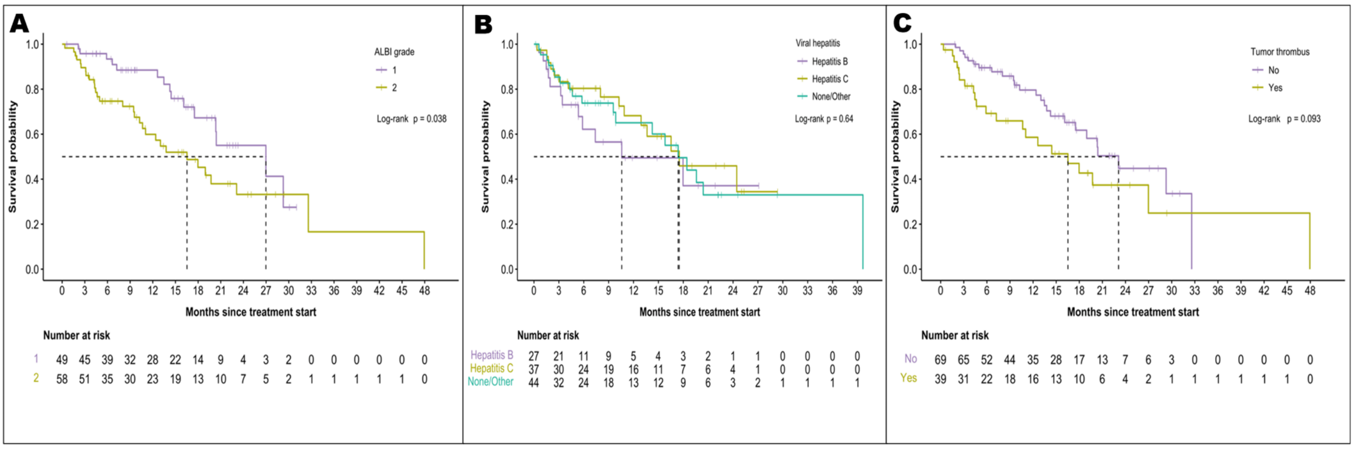

Figure S1: Overall Survival in Patients Treated with Atezolizumab with Bevacizumab Stratified by (**A**) ALBI grade (**B**) Liver etiology and (**C**) Portal vein tumor thrombosis

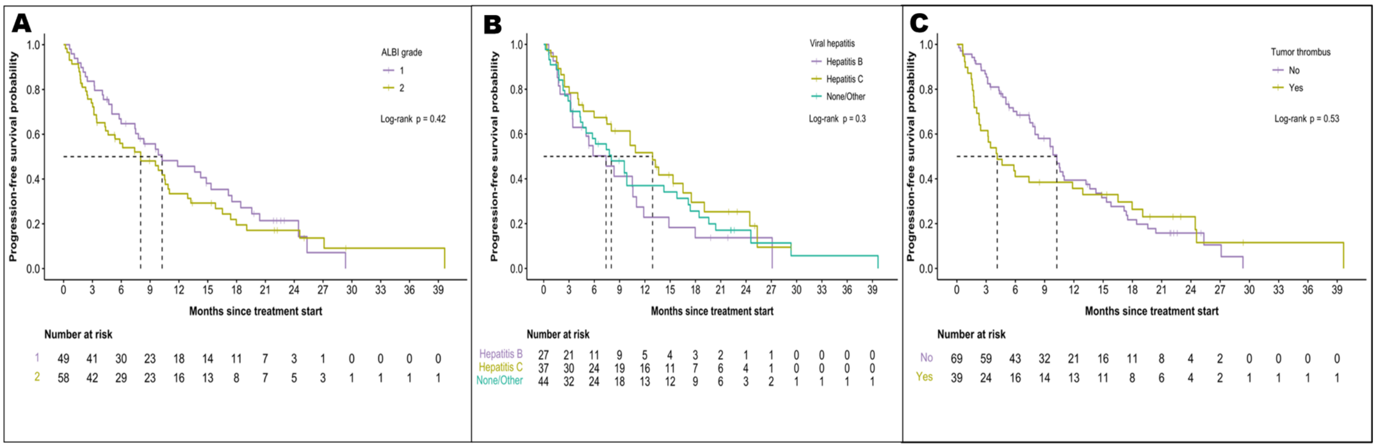

Figure S2: Progression-Free Survival in Patients Treated with Atezolizumab with Bevacizumab Stratified by **A)** ALBI grade **B)** Liver etiology and **C)** Portal vein tumor thrombosis

Table S1: Impact of clinical characteristics on bleeding risk.

		Bleeding events		
**Characteristic**	**Total included**	**No (n = 95)**	**Yes (n= 17)**	**p-value**
**Cirrhosis**	75 (67.0%)	64 (67.4%)	11 (64.7%)	0.83
**Hepatitis B**	28 (25.0%)	20 (21.1%)	8 (47.1%)	**0.03**
**Hepatitis C**	37 (33.0%)	32 (33.7%)	5 (29.4%)	0.73
**NASH**	17 (15.2%)	13 (13.7%)	4 (23.5%)	0.29
**Excess alcohol use**	25 (22.3%)	24 (25.3%)	1 (5.9%)	0.11
**Prior locoregional treatment**				
**TACE procedures**	29			
0	83 (74.1%)	71 (74.7%)	12 (70.6%)	
1	11 (9.8%)	10 (10.5%)	1 (5.9%)	1.00
2	14 (12.5%)	13 (13.7%)	1 (5.9%)	0.72
3	4 (3.6%)	1 (1.1%)	3 (17.6%)	**0.03**
**Radiofrequency Ablation**	33 (29.5%)	28 (29.5%)	5 (29.4%)	1.00
**Stereotactic Body Radiation Therapy**	17 (21.8%)	15 (23.1%)	2 (15.4%)	0.72
**Liver resection**	29 (25.9%)	22 (23.2%)	7 (41.2%)	0.14
**Liver transplant**	1 (0.9%)	0 (0.0%)	1 (5.9%)	0.15
**Macrovascular invasion**	41 (36.9%)	35 (37.2%)	6 (35.3%)	0.88
Unknown	1	1	0	
**Tumor size**	96			0.56
Mean ± SD	5.8 ± 4.7	5.7 ± 4.8	6.0 ± 4.3	
Median (Range)	3.8 (0.0, 18.0)	3.8 (0.0, 18.0)	6.0 (0.0, 15.6)	
Unknown	16	16	0	
**Tumor Histology**	76			**0.04**
HCC	73 (96.1%)	66 (98.5%)	7 (77.8%)	
Mixed CCA-HCC	3 (3.9%)	1 (1.5%)	2 (22.2%)	
Unknown	36	28	8	

NASH: non-alcoholic steatohepatitis; TACE: Transarterial Chemoembolization; SD: standard deviation; HCC: hepatocellular carcinoma; CCA: cholangiocarcinoma.
